# 

**Published:** 1907-05

**Authors:** 


Vol. XXII. MAY, 1907. No. 11.

### TEXAS MEDICAL JOURNAL

"RED BACK"

PUBLISHED AT AUSTIN, TEXAS, BY

F. E. DANIEL, M. D., Proprietor

PRINTED BY THE VON BOECKMANN-JONES CO.

Subscription, $1.00 a Year, in Advance. - - - Single Copies, 10 Cents

Entered at the Postoffice at Austin, Texas, as Second-class Mail Matter

